# A Qualitative Examination of the Content Validity of the EQ‐5D‐5L and EQ‐5D‐Y‐3L in Adult and Paediatric Patients With Duchenne Muscular Dystrophy

**DOI:** 10.1111/hex.70431

**Published:** 2025-09-10

**Authors:** Richard Huan Xu, Rui Jiang, Chenxi Yang, Dong Dong

**Affiliations:** ^1^ Department of Rehabilitation Sciences, Faculty of Health and Social Sciences Hong Kong Polytechnic University Hong Kong China; ^2^ Shenzhen Research Institute The Chinese University of Hong Kong Shenzhen China; ^3^ JC School of Public Health and Primary Care The Chinese University of Hong Kong Hong Kong China

**Keywords:** content validity, Duchenne muscular dystrophy, EQ‐5D, EQ‐5D‐Y, qualitative study

## Abstract

**Objective:**

This study examined the content validity of EQ‐5D for Chinese patients with DMD. Specifically, it investigated: (1) the content validity of EQ‐5D‐5L in adult DMD patients and (2) the content validity of EQ‐5D‐Y‐3L (Y‐3L) in DMD patients aged 8–15 years.

**Method:**

This qualitative study used semi‐structured interviews and conducted one‐on‐one and online, with two groups of individuals with DMD to examine the content validity of the EQ‐5D‐5L and Y‐3L. All participants were recruited from a nationwide DMD patient association. The study included both adult and paediatric DMD patients. Data were analysed using a framework approach.

**Results:**

A total of 30 interviews were completed, with 15 for adult patients and 15 for paediatric patients. Overall, EQ‐5D‐5L and Y‐3L covered the most important aspects of HRQoL for DMD patients. However, many adult patients were unclear about the ‘Mobility’ item, suggesting clearer guidance on whether it refers to unaided physical mobility or mobility with assistive devices like wheelchairs. Paediatric patients felt that the three option levels were not enough to accurately describe their mobility. Both adult and paediatric patients noted that essential activities like ‘toileting’ were missing from the self‐care section. They also found the examples of usual activities, such as housework, to be unsuitable. Some paediatric patients disliked the examples in Y‐3L (headaches and itchiness) and preferred having no examples at all. Regarding mental health questions, both groups struggled to connect their condition with psychological effects.

**Conclusion:**

Both EQ‐5D‐5L and Y‐3L showed acceptable content validity; however, our findings indicated the need to refine and optimise these tools to better capture critical HRQoL domains for DMD and potentially other neuromuscular diseases.

**Patient or Public Contribution:**

The study was conducted in partnership with a DMD patient association, which played a key role in shaping the study's design and implementation. The association also contributed to reviewing the interview schedule.

## 1 Introduction

Duchenne muscular dystrophy (DMD) is a rare X‐linked genetic neuromuscular condition, which occurs primarily in males, and the main symptoms are difficulties with climbing stairs, toe walking and frequent falls [1]. Most patients with DMD succumb to cardiac or respiratory failure in their late teens or 20s [2]. Assessing health‐related quality of life (HRQoL) in patients with DMD is crucial because it provides a comprehensive understanding of the disease's impact beyond physical symptoms, capturing emotional, social and functional dimensions in a population with a progressive, life‐limiting condition. As new treatments emerge, robust HRQoL measures grounded in validated tools enable clinicians and researchers to evaluate treatment effectiveness and guide patient‐centred care decisions. This is particularly vital in rare diseases (RDs) like DMD, where patient scarcity complicates research, making accurate HRQoL assessment essential for optimising therapeutic outcomes and improving quality of life for patients and their families.

Studies measuring HRQoL in patients with DMD show considerable variation, indicating challenges in assessing HRQoL using common generic patient‐reported outcome measures (PROMs) in this rare and unique population [3]. The EQ‐5D, the most widely used generic preference‐based measure worldwide, demonstrates robust psychometric properties and has been used to support cost–utility analysis (CUA) across various patient groups. However, evidence supporting its appropriateness for DMD patients in CUAs remains limited. Addressing this gap by evaluating the content validity of the EQ‐5D is critical to ensure its appropriateness for capturing HRQoL in DMD, thereby enhancing the accuracy of health economic evaluations for this population.

Content validity, which examines the construct of interest within a target population and context, is the first step in assessing the validity of a PROM. It is fundamental to a PROM's usefulness in measuring health status and may affect all other measurement properties [4, 5].

Limited evidence exists regarding the content and structural validity of PROMs assessing HRQoL in DMD. A review found low‐quality evidence about the content validity of EQ‐5D [5], highlighting the need for further research. Additionally, the content validity of the newly developed DMD‐specific preference‐based measure, DMD‐QoL‐8D, remains unreported, and its psychometric properties have not been compared with the EQ‐5D‐5L [6]. A recent study assessed the usefulness of EQ‐5D‐3L to measure HRQoL in patients with DMD and provide preliminary evidence about the appropriateness of the EQ‐5D‐3L in patients with DMD [7]. However, another study criticised its methodology, noting an unclear description of content validity assessment and emphasising the need for a comprehensive evaluation [8]. It is vital to clarify the appropriateness of EQ‐5D in DMD, addressing methodological concerns and advancing PROM development for this population.

Currently, the NICE's methods review report about HRQoL acknowledges insufficient evidence for measuring HRQoL in RDs using the EQ‐5D [9]. A key conclusion is that the evaluation of the appropriateness (content validity) of the EQ‐5D and the other condition‐specific PROMs in RDs should be prioritised. Moreover, the third patient‐focused drug development guidance from the US FDA [10] emphasises that psychometric properties of a PROM should not be assessed until its content validity is established, particularly in RD research where patient scarcity complicates such assessments. In recent years, several new treatments for DMD, including gene therapies (e.g., Elevidys) and exon‐skipping drugs (e.g., Exondys 51), have emerged. To support their evaluation, empirical evidence prioritising content validity assessment is essential, establishing a robust foundation for accurate HRQoL measurement in DMD.

HRQoL is increasingly recognised as an important outcome in RDs [11]. Given reported concerns about the unclear content validity of EQ‐5D across various populations [12, 13], addressing the knowledge gap regarding its content validity in assessing HRQoL in DMD is crucial. Thus, this study aimed to examine the content validity of EQ‐5D in Chinese patients with DMD. Specifically, we assessed: (1) the content validity of EQ‐5D‐5L in adult patients with DMD and (2) the content validity of EQ‐5D‐Y‐3L (Y‐3L) in children and adolescents with DMD aged 8–15 years. By assessing content validity in this specific population, this study contributes to ensuring that HRQoL measures are relevant and effective for DMD patients, supporting better clinical and economic decision‐making.

## 2 Methods

### 2.1 Study Design

This qualitative study involved semi‐structured, one‐on‐one and web‐based interviews with two groups of individuals with DMD. We opted for one‐on‐one interviews for several reasons. First, previous studies evaluating the content validity of PROMs have demonstrated that one‐on‐one interviews allow for deeper, more individualised insights into patients' experiences and perceptions. These studies highlight that one‐on‐one formats better capture nuanced responses, ensuring the instrument's relevance and clarity are thoroughly evaluated. Second, the poor and progressive health condition of DMD patients, characterised by severe mobility limitations and fatigue, poses significant logistical challenges for convening focus groups. Co‐ordinating a suitable time for multiple patients with such constraints is often impractical, and group settings may exacerbate fatigue or discomfort, potentially compromising data quality. Additionally, one‐on‐one interviews provide a safer, more comfortable environment for patients to express sensitive or personal perspectives on their HRQoL, which may be inhibited in a group dynamic due to social pressures or varying disease stages among participants. Finally, individual interviews allow for tailored pacing and accommodations, such as breaks or adaptive communication methods, which are critical for paediatric and adult DMD patients with diverse physical and cognitive needs.

### 2.2 Participants

The first targeted population was the adult patients diagnosed with DMD, and the second targeted population is the paediatric patients with DMD aged between 8 and 15 years. The content validity of the EQ‐5D‐5L and Y‐3L was examined in the first and second types of participants, respectively. In this study, the inclusion criteria for adult patients with DMD were (1) aged 18 years or older; (2) male patients; (3) no cognitive problems and (4) ability to provide informed consent. For paediatric patients with DMD, the inclusion criteria were (1) aged between 8 and 15 years; (2) male patients and (3) legal guardians (parents, grandparents or paid caregivers) could provide informed consent. Although all members of the patient organisation were patients with DMD formally diagnosed, a diagnosis certificate issued by a specialist needed to be presented to the research team to confirm their eligibility. Two groups of DMD patients (ambulatory and non‐ambulatory) were recruited for two types of participants (adult and child), respectively. A one‐item question was used to dichotomise participants into two groups. Those who could walk independently outdoors for at least short distances were defined as ‘ambulatory’ participants, and those who could not walk outdoors without help were defined as the ‘non‐ambulatory’ group.

All participants were recruited from the *Beijing Dear Mom & Dad DMD patient centre*, one of the largest DMD patient organisations in China. Members consisted of DMD patients or their caregivers. For our recruitment, the organisation's managers informed participants about all aspects of the study through its internal social network. Interested participants' information was then sent to the research team for initial screening and contact. Study information and consent forms were sent via email or WeChat (a multi‐purpose social media app) to patients or caregivers who met the eligibility criteria. If a participant agreed to join the study and provided informed consent, the lead researcher contacted them to schedule the interview. Once confirmed, an interview lasting around 45–60 min was conducted at the participant's convenience. For paediatric DMD patients, the primary caregiver was invited into the chat room but was not allowed to participate in the interview.

The interview process was concluded once data saturation was achieved in each of the adult and paediatric groups. Saturation was determined using a single, widely accepted criterion: no new codes, themes or insights emerged from additional interviews, indicating that the data were sufficiently comprehensive to address the research objectives [14]. This was assessed separately for each group to ensure that the unique perspectives of adult and paediatric patients were fully captured. After conducting 15 interviews per group, we determined that saturation had been reached in both the adult and paediatric cohorts, as no further novel insights or perspectives were identified, and the data adequately supported the development of robust themes and concepts.

### 2.3 Interview Procedure

Interviews were conducted using a semi‐structured guide, designed through literature review and expert consultation to assess the content validity of the EQ‐5D for patients with DMD. The interview guideline is presented in the Appendix (Tables a1 and a2). The interview process consisted of three phases: First, understanding the interviewees' background, which included their socio‐economic status, health condition, DMD diagnosis and treatment. Second, concept elicitation. Participants were asked open‐ended questions (e.g., How does DMD or its treatment impact your quality of life?) about how DMD affects their health/quality of life, without interviewers suggesting possible impact domains. In the third phase, participants were asked to complete the EQ‐5D‐5L or Y‐3L, followed by a cognitive interview. All interviewees were provided with operational definitions for each EQ‐5D‐5L/Y dimension to facilitate reflection on their health within these domains during the interviews (Tables a3 and a4). This evaluated their comprehension of the instrument's instructions, items and response options, as well as its relevance and completeness (e.g., Does DMD impact your quality of life in any other ways that are not captured by this questionnaire?). To minimise order bias, the sequence of EQ‐5D items discussed varied between interviews. The Institutional Review Board of Hong Kong Polytechnic University approved the research protocol (Ref. ID: HSEARS20230412007). All the participants provided written informed consent.

### 2.4 Measures

#### 2.4.1 EQ‐5D‐5L

The EQ‐5D‐5L is a generic, preference‐based HRQoL instrument. Its health state classification system consists of five items (mobility, self‐care, usual activities, pain/discomfort and anxiety/depression) with five response levels (from no problem to extreme problems) to describe an individual's health state. It also has a visual analogue scale (EQ VAS), which asks participants to rate their current health from 0 (the worst health you can imagine) to 100 (the best health you can imagine) [15].

#### 2.4.2 Y‐3L

The Y‐3L is a child‐friendly EQ‐5D version. Its descriptive system has five dimensions (mobility; looking after myself; doing usual activities; having pain or discomfort; and feeling worried, sad or unhappy) with five (no problems, a little bit of problems, some problems, a lot of problems and cannot/extreme problems) response levels. The Chinese version of the Y‐3L has been well translated and used in several patient groups [16].

### 2.5 Data Analysis

The interviews were divided into two distinct sections: the first explored participants' understanding of health, while the second evaluated the relevance and clarity of the descriptive system. Each section was analysed independently using a structured framework approach [17]. All interviews were audio‐recorded, transcribed verbatim and analysed using Dedoose software. Two researchers independently coded the transcripts, assigning descriptive codes to segments of text that captured specific ideas, experiences or phenomena related to the content validity of the EQ‐5D‐5L and EQ‐5D‐Y‐3L. Initial codes were developed based on the interview guide and literature review, with additional codes added iteratively to capture emerging ideas from the data. The researchers met regularly to compare coding, resolve discrepancies through discussion, and, when necessary, consulted a third researcher to reach consensus. Related codes were then grouped into themes, which represented broader patterns or categories of meaning relevant to the research objectives (e.g., limitations in the mobility dimension). Themes were refined through iterative review of the transcripts to ensure they accurately reflected participants' experiences. The analysis for the EQ‐5D‐5L (adult patients) and EQ‐5D‐Y‐3L (paediatric patients) was conducted separately to account for differences in the instruments and populations. The coding tree is presented in Figure a1.

## 3 Results

### 3.1 Participant's Background Characteristics

Table 1 presents the background characteristics of interviewees. Each group, both adult and paediatric patients, consisted of 15 male participants. Among adults, 53% had attained a college or higher degree, while 60% of paediatric patients had completed primary school education. Both groups had more urban than rural patients, with most perceiving their family income as average level. The mean age for adults was 21 years (range: 18–32), and for paediatric patients it was 11.9 years (range: 8–14).

**Table 1:** Participants' background characteristics.

	Adult sample		Paediatric sample	
	*n*	%	*n*	%
Sex				
Male	15	100	15	100
Educational level				
Primary school	2	13.3	9	60
Junior school	1	6.7	6	40
Senior school	4	26.7	0	0
College or above	8	53.3	0	0
Family registry				
Urban	11	73.3	9	60
Rural	4	26.7	6	40
Perceived family income				
Lower than local average	4	26.7	2	13.3
Equal to local average	10	66.7	12	80
Higher than local average	1	6.6	1	6.7
Overall health status				
Very good				
Good	4	26.7	5	33.3
Normal	8	53.3	9	60
Bad	1	6.7	0	0
Very bad	2	13.3	1	6.7
Type				
Ambulatory	1	6.7	10	66.7
Non‐ambulatory	14	93.3	5	33.3
	Mean (range)	SD	Mean (range)	SD
Age	21 (18–32)	3.8	11.9 (8–14)	1.98

### 3.2 Comprehensiveness of EQ‐5D‐5L Dimensions

The EQ‐5D‐5L was generally considered adequate for measuring health status and HRQoL among adult patients with DMD. However, participants identified limitations in its capability, highlighting the need for greater specificity to capture DMD‐related experience. The following themes and sub‐themes emerged from the qualitative analysis, as presented in Table 2.

**Table 2:** The comments on EQ‐5D‐5L from adult DMD patients.

**Dimensions**	**Perceived points**	**Comments from adult patients**
Holistic perception	Need more details	I think all five questions on this questionnaire share a common issue: their options and descriptions are too general and lack specificity. This aspect may need reconsideration. While the form's structure is comprehensive, some minor details could benefit from further refinement to enhance clarity and precision (A‐14) These five questions indeed address the main topic of health, but they lack detail. Overall, this scale shouldn't pose any problems because it only covers five major directions, but, as we mentioned. You can't extract substantial information from these five questions alone (A‐02)
Mobility	Need a precise definition	It's like when I use an electric wheelchair, I can move around freely (A‐06) We should consider adding a point about mobility aids in the first item. This would account for varying levels of mobility among disabled individuals, including those who can move independently and those who rely on wheelchairs or other assistive devices. This addition would provide a more comprehensive and sensitive evaluation (A‐11)
	Add upper limb strength	Mobility encompasses more than just walking; it includes upper limb strength, such as the ability to pick up objects. We should consider mobility from both upper and lower limb perspectives, especially in cases of DMD. In DMD, the strength of the upper and lower limbs can vary significantly (A‐07)
	Engage in mild physical exercise	Are you engaging in appropriate exercise? This could also be considered one. It would be better to ask, ‘To what extent am I able to exercise?’ This is more suitable than mobility alone (A‐11)
Self‐care	‘Washing or dressing myself’ is too narrow	I think self‐care shouldn't be limited to just bathing or dressing; it can be extended to other aspects as well. For example, using the toilet and eating (A‐07) Just bathing or dressing alone is too limited. Self‐care, I feel, is much more complex than that. Like you just asked me about turning over in bed, getting up from bed, and issues with personal hygiene, right? Simply bathing or dressing might not be enough (A‐13)
	‘Self‐care’ is unclear	In terms of self‐care, we could separate out another category, such as feeding oneself. This is because washing and dressing might have a different level of difficulty compared to eating by oneself. I don't have much feeling about the others, as most of them are done with parental help. There's probably little that one can do independently (A‐01) Self‐care activities vary in difficulty based on their nature. Stationary tasks like grooming, face washing and teeth brushing are simpler as they can be done in one place. Activities requiring movement, such as using the toilet or bathing, are more challenging due to the need for transferring or moving around. Thus, self‐care can be categorised into two types: easier stationary tasks and more difficult mobility‐related tasks (A‐14)
Usual activities	Add social life activities	I think communicating with others is quite important for usual activities … at least you need to have someone to talk to, right? If you don't have anyone to communicate with, it feels particularly meaningless (A‐01) My personal suggestion is to divide it (usual activities) into two parts. That is, individual and team. It means doing some things by yourself, and doing some things with others. But if we combine these two questions, overall, it feels just about the impact on the individual (A‐02) Let's add ‘go out’! For transportation, now we can basically only use private cars. Public transportation is not feasible (A‐15)
	Current examples are challenging	The item is difficulty to select. I can only say that if I were to complete them (usual activities) on my own, I could only manage the leisure aspects. The usual activities mentioned above include things like work and study, household chores, or leisure activities. Work and household chores are impossible for me to accomplish. As for leisure activities, apart from computer‐related things, I can't do anything else (A‐03) The issues mentioned above are all related to some abilities in daily life, but for most of them, it's either very difficult for me to do alone or I can't complete them by myself at all. These daily activities are extremely challenging for me (A‐04)
	Diverse ranges in activity difficulties	(Things we do regularly) do indeed have differences. For household chores, the difficulty is certainly greater, so we basically don't do these kinds of activities. For leisure activities, the difficulty is a bit less because you can adjust according to your own situation. As for studying and working, it depends on which aspect we're talking about (A‐08) Different activities have different levels of difficulty. So if we just broadly categorise it as ‘usual activities’, and then you mention so many different things afterwards, how can one choose, right? What if some people have very little difficulty with studying, but find housework extremely difficult? That would be very hard to deal with (A‐14)
Pain/discomfort	Unmeaningful option levels and an unclear source of pain	This pain still persists in my body, it hasn't gone away (A‐02) Regarding this pain or discomfort, I feel that ‘very severe’ and ‘severe’ are pretty much the same (A‐06) Pain or discomfort. I would say I have moderate pain and discomfort, because of sitting for long periods, which over time has led to stomach pain (A‐03)
Depression/anxiety	Overlooked loneliness and unique psychological challenges	I feel that there are too few items related to mental health. In fact, I think that for people like us who have this disease or disability, there are many psychological aspects that are different from others. For example, it's not just anxiety and depression, but also other emotions like loneliness (A‐06) …You know, those patients who are younger than me, their psychological issues might be more serious. It is not only about depression or anxiety, they might be very conflicted about their future development or their hopes… (A‐14) There's another issue, which is that vague adjectives like ‘slight’, ‘moderate’, ‘severe’ and ‘very severe’ (for option levels) are not easy to understand (A‐14)
Supplement	Consider social support and respect	Moreover, social support is very important. Without social support, one can feel quite lonely with few friends, leading to a sense of isolation. This is because some individuals haven't attended much schooling from a young age, resulting in a lack of social support, with fewer classmates and teachers in their lives (A‐03) It's about making him feel more respected and valued, which is different from a situation where he feels like he has no choice but to do something (A‐07)

#### 3.2.1 Mobility Theme

Adult participants identified limitations in the mobility dimension of the EQ‐5D‐5L, highlighting three key sub‐themes—Need for a precise definition: Participants noted ambiguity in the term ‘mobility’, questioning whether it referred to unaided movement or included assistive devices like wheelchairs. Inclusion of upper limb strength: Participants emphasised that mobility should encompass upper limb function, as disparities between upper and lower limb strength are significant in DMD. Relevance of mild physical exercise: Participants suggested that the ability to engage in mild exercise should be considered a key indicator of mobility.

#### 3.2.2 Self‐Care Theme

The self‐care dimension was seen as too narrow, with participants advocating for a broader range of activities to reflect the complex needs of DMD patients. Two sub‐themes were identified: First, the limited scope of ‘washing or dressing myself’. Participants felt that self‐care should extend beyond washing and dressing to include tasks like using the toilet, eating and getting out of bed. Second, ambiguity in the term ‘self‐care’. The term ‘self‐care’ was unclear, as most participants required caregiver assistance, yet some could perform tasks with support. Participants highlighted varying levels of difficulty, with stationary tasks (e.g., grooming) being easier than mobility‐related tasks (e.g., using the toilet).

#### 3.2.3 Usual Activities Theme

Participants identified the need to refine the examples of usual activities to better reflect the experiences and challenges of adults with DMD. The first sub‐theme is the ‘importance of social life activities’. Participants emphasised the inclusion of social activities, such as communicating with others and going out, as central to their quality of life. The second sub‐theme is ‘challenges with current examples’. The examples provided (e.g., work and housework) were often perceived as too challenging or irrelevant. The third sub‐theme is ‘variability in activity difficulty’. Participants highlighted the diverse difficulty levels of usual activities, complicating the selection of appropriate response options.

#### 3.2.4 Pain/Discomfort Theme

Participants reported issues with the pain/discomfort dimension, particularly its response options and ambiguity about the source of pain. The first identified sub‐theme is ‘unmeaningful response levels’. The first three response levels were considered irrelevant due to the severe, chronic pain experienced by most DMD patients. Second, ‘unclear source of pain’. Participants were uncertain whether pain stemmed from DMD, treatment side effects or lifestyle factors.

#### 3.2.5 Depression/Anxiety Theme

The depression/anxiety dimension was seen as insufficient in capturing the full range of psychological challenges faced by DMD patients. The first sub‐theme is ‘overlooked loneliness and psychological challenges’. Participants highlighted emotions like loneliness and broader psychological issues beyond anxiety and depression. Another sub‐theme is ‘ambiguity in response levels’. The adjectives used for response levels (e.g., ‘slight’ and ‘moderate’) were seen as vague.

#### 3.2.6 Supplement to the EQ‐5D‐5L Descriptive System

Two additional themes emerged regarding potential improvements to the EQ‐5D‐5L. The first is the ‘longer time frame’. Participants suggested using a longer time frame (e.g., ‘over the past few weeks’) to better capture the progressive nature of DMD, rather than focusing on ‘today’. Another one is ‘inclusion of social support and respect’. Participants emphasised the importance of social support and respect as critical dimensions for HRQoL.

### 3.3 The Comprehensiveness of Y‐3L Dimensions

Paediatric patients provided generally positive feedback on the clarity, comprehensibility and relevance of the EQ‐5D‐Y‐3L, but they identified areas for improvement to better address DMD‐specific experiences. The following themes and sub‐themes emerged, as presented in Table 3.

**Table 3:** The comments on EQ‐5D‐Y‐3L from DMD children.

**Dimensions**	**Perceived points**	**Comments from DMD children**
Mobility	Add more examples	There's a big difference between walking on flat ground and on stairs. I can actually go upstairs on my own if I lean against something, but it's quite tiring and dangerous. So now, when going up stairs, I almost always need someone beside me to push me up. I can go downstairs by myself (C‐013) (DMD) limits my mobility. For example, other children can run very fast, but I run very slowly. It also limits my ability to climb stairs; it's very strenuous for me to go upstairs (C‐03) Distance is an important issue, particularly in terms of mobility. It refers to how far a person can walk on their own. In my case, I can walk about 2 km by myself (C‐014) The questionnaire lacks an item about ascending and descending stairs. It also omits other exercises or sports. The focus is solely on walking, neglecting other activities. There's a significant difference between navigating stairs and moving on level ground (C‐015)
Looking after myself	Add more activities	Brushing teeth and washing face were not mentioned, I can wash up by myself (C‐05) Taking medicine daily wasn't mentioned, my mom prepares the medicine for me, and she puts some hot water in the cup (C‐06) It's not just bathing and dressing, but also eating and using the toilet. Except for eating, I can't do any of the others at all (C‐09) This question didn't mention using the toilet (C‐010) I feel that self‐care surely isn't just about bathing and dressing, it also includes what we just mentioned about going to the toilet (C‐013) Eating wasn't mentioned. Does using the toilet count as self‐care? It's very difficult to squat and stand up when using the toilet, sitting down is a bit easier than standing up. These things have different levels of difficulty (Mom: The most difficult is using the toilet, it's hardest when falling. Getting up after using the toilet is the most difficult. The easiest to say is that movement is not good. Bathing is somewhat difficult.) (C‐015) Then I feel that, how should I put it, whether or not sleep quality at night can also be included in self‐care, I think, but this might be a bit of a stretch (C‐013)
Doing usual activities	Accurately represent their situation	The question is quite comprehensive, because it already includes school, interests, activities, sports, play and current study life. This is quite three‐dimensional and complete (C‐013) (Several different activities, including school interests, activities, sports, play and things done with friends and family) The level of difficulty is about the same. There's nothing to add (C‐014)
	Exercise seen as distinct	…I still play games with friends for entertainment, but I can't participate in other activities like going out for sports or playing ball games. Even if I go, they say it's quite dangerous and that I'd better not come (C‐013) There are some differences in schooling, hobbies, sports, play, and things done with family and friends…. It's easier to play with friends, however, things related to sports are a bit difficult (C‐011) Using hands to lift things, asking someone to grab something, and bending down to pick up things (C‐03) (Among regular activities like schooling, hobbies, activities, sports, play, and things done with family and friends) going to the toilet is quite difficult. Additionally, when playing with classmates, sometimes they don't notice, and suddenly someone comes out and knocks me down. It's easier when playing indoor games together … furthermore, eating is also easy (C‐10)
Pain and discomfort	Pain cases of leg, muscle and sleep‐related discomfort	Sometimes even when I'm not moving, my legs suddenly hurt a lot (C‐07) Actually, every moment of every day, my whole body is in pain, but it's tolerable, generally speaking. Bones and flesh (pain) (C‐13) At night when sleeping, if I don't turn over, it feels a bit uncomfortable (C‐08)
Feeling worried, sad or unhappy	Ask about the timing and causes	Add how you feel now, and how you generally felt before, or how you've generally always been? This should probably be asked separately (C‐13) What things make you feel happy or unhappy? (C‐14) (Omission) A bit quick to anger (C‐15)
Supplement	Add questions (muscle strength‐related activities, DMD‐friendly facilities, sleep, treatment and schooling impact)	(These issues are very important in everyday life, but weren't addressed in the questions I just saw.) Bringing up even a few points feels somewhat challenging (C‐03) At 14, he stopped attending junior high school, mainly because of the elevator issue—the school had no elevators. As a result, his life became very unhappy (C‐01) I think sleep quality can be included in self‐care, but it might be better to list it as a separate item if possible (C‐13) I think there's an important question, ‘Are you currently in school?’ Because many people with our condition don't attend school, as is the case with my two friends (C‐01) Taking medication is an important daily task (yet missed) (C‐06)

#### 3.3.1 Mobility Theme

Participants suggested enhancements to the mobility dimension to better reflect the specific challenges faced by children with DMD. The first sub‐theme is ‘need for additional examples’.

Participants recommended including examples like navigating stairs and clarifying whether mobility encompasses arm movements or assistance needs. The second sub‐theme is ‘importance of distance and assistance’. Participants highlighted the relevance of walking distance and the need for assistance.

#### 3.3.2 Looking After Myself Theme

The self‐care dimension was perceived as too narrow, with participants advocating for a broader range of activities. One sub‐theme is ‘inclusion of additional activities’. Participants suggested adding activities like using the toilet, eating, taking medication and improving sleep quality. Another sub‐theme is ‘variability in task difficulty’. Participants highlighted varying levels of difficulty for self‐care tasks, with using the toilet being particularly challenging.

#### 3.3.3 Doing Usual Activities Theme

Participants provided mixed feedback on the examples of usual activities, noting both their relevance and limitations. Sub‐theme *1, ‘r*elevance of examples’*.* Some participants felt the examples (e.g., school, sports and play) accurately reflected their experiences. Sub‐theme 2, ‘exercise as a distinct activity’. Participants viewed exercise as distinct due to its unique challenges in DMD.

#### 3.3.4 Having Pain or Discomfort Theme

Participants reported varied experiences with pain and discomfort, with some noting its absence and others describing specific issues. While some reported little to no pain, others described leg pain, muscle pain or sleep‐related discomfort.

#### 3.3.5 Feeling Worried, Sad or Unhappy

Participants suggested refinements to the emotional dimension to capture the timing and causes of emotions. Sub‐theme: Importance of timing and causes. Participants emphasised the need to ask about the timing and causes of emotions to provide a more nuanced understanding. Sub‐theme: Inclusion of irritability. One participant suggested adding a question about irritability, noting, ‘A bit quick to anger’, potentially linked to medication side effects.

#### 3.3.6 Supplement to the Y‐3L Descriptive System

Participants proposed additional items to enhance the relevance of the EQ‐5D‐Y‐3L for DMD. Suggestions included questions about muscle strength‐related activities, DMD‐friendly facilities, sleep quality, treatment impacts and schooling.

## 4 Discussion

This study is the first to examine the content validity of the EQ‐5D instrument for patients with DMD. We conducted interviews with adult and paediatric DMD patients to assess the suitability of the EQ‐5D instruments for measuring HRQoL in this population. The findings indicated that patients generally satisfied the conciseness of EQ‐5D‐5L and EQ‐5D‐Y, recognising their effectiveness as generic instruments in capturing the key aspects of HRQoL. However, the study identified limitations in the EQ‐5D's applicability to DMD and potentially other rare musculoskeletal diseases. The mobility dimension is inadequately defined and ambiguous for most DMD patients, who often lack ambulatory function, which may introduce measurement bias. Additionally, the term ‘uncomfortable’ lacks precision in its intensity, scope or focus, resulting in inconsistent patient interpretations. Furthermore, the absence of a social relationships dimension, particularly critical for paediatric patients, overlooks a vital component of their HRQoL. These findings suggest that, while the EQ‐5D remains a valuable generic instrument for assessing HRQoL across diverse populations, it may require refinement to effectively measure HRQoL in RDs like DMD, where health challenges differ significantly from those of the general population.

The majority of adults with DMD face profound mobility limitations, with most unable to ambulate independently, even when using wheelchairs, which provide only a modest degree of independent movement [18]. Consequently, the ‘mobility’ item in the EQ‐5D instrument lacks relevance as a meaningful metric for assessing HRQoL or functional capacity in adult DMD patients without a precise definition. This aligns with previous findings that while the mobility item is relevant for general health, it lacks specificity for certain conditions, including DMD [19, 20, 21]. Conventional mobility measures fail to capture the lived experiences and daily functioning of DMD patients, for whom mobility extends beyond walking to include activities such as running or other physical tasks, as highlighted in a UK study involving children with various health conditions [22]. Although similar observations have been reported in studies of movement disorders, this is the first study to address this issue specifically in patients with RDs. To improve the applicability of the EQ‐5D for DMD and other neuromuscular patients, the mobility dimension should be clearly defined to encompass assisted movement (e.g., using a wheelchair), ensuring it better reflects their lived reality.

The EQ‐5D's pain/discomfort item presents challenges in assessing HRQoL for patients with DMD, as its ambiguity has been widely debated in prior research [22]. Our findings align partially with studies that identify pain and discomfort as a critical factor affecting HRQoL in DMD patients. However, DMD often cause widespread, high‐intensity chronic pain, which most patients have endured for nearly their entire lives. This prolonged exposure increases their pain tolerance, making it difficult for them to accurately select a pain level on the item. Previous studies have reported that this item is susceptible to measurement errors, such as under‐reporting and inconsistent responses [23, 24], due to its vague terminology and response options [25]. Furthermore, patients often find the terms ‘pain’ and ‘discomfort’ unclear, a concern echoed in previous studies [24, 26]. Our study confirms that DMD patients struggle to interpret whether the item refers to physical pain from the disease itself or psychological discomfort, such as a feeling of powerlessness in managing their conditions [23]. Additionally, patients expressed uncertainty about the timing of the assessment, questioning whether it pertained to pain before or after treatment, as these experiences can differ significantly. Although interviewees generally understood the item, they indicated its lack of conceptual clarity. This ambiguity may reduce the responsiveness of the pain/discomfort item in clinical settings for DMD management. Providing clear examples or separating them into two items to distinguish between physical pain and psychological discomfort, tailored to those with chronic, high‐intensity pain, could enhance the accuracy and relevance of the EQ‐5D in assessing HRQoL for DMD patients.

Social relationships are a critical domain strongly linked to HRQoL and overall well‐being for most RD patients [27]. In this study, we found that insufficient social interactions with peers can negatively impact physical and mental health, contributing to increased stress and social isolation for DMD patients. Empirical and theoretical evidence strongly support incorporating social relationships as a bolt‐on dimension for the EQ‐5D [28, 29, 30]. This dimension would capture the understanding, empathy and encouragement offered by friends, family and support groups, which are essential for helping DMD patients manage stress and anxiety. Currently, the EQ‐5D lacks a direct measure of social relationships [28], despite their alignment with the WHO's definition of health, which encompasses physical, mental and social well‐being. For paediatric patients, social relationships are particularly important, as peer support fosters shared experiences and coping strategies that enhance their well‐being [31]. Including social relationships as a bolt‐on dimension would enhance the EQ‐5D's sensitivity to these critical social dynamics. This is particularly relevant given that other HRQoL instruments, such as the SF‐6D [32] and PedsQL [33], already include social functioning as a core component, highlighting the gap in the EQ‐5D's descriptive system.

### 4.1 Recommendations and Future Research Direction

To improve the EQ‐5D's performance for DMD, we propose the following research directions based on our findings: First, conduct a comprehensive psychometric evaluation of the EQ‐5D in DMD patients, testing several hypotheses derived from qualitative findings. Relevance of dimensions (Hypothesis 1): the EQ‐5D dimensions effectively capture HRQoL aspects critical to DMD patients. Comprehensibility (Hypothesis 2): the EQ‐5D dimensions are clear and suitable for DMD patients' physical and cognitive abilities. Sensitivity to change (Hypothesis 3): the EQ‐5D detects changes in DMD patients' health status over time, reflecting both deterioration and improvements. Impact of disease progression (Hypothesis 4): the EQ‐5D scores correlate with the stages of disease progression in DMD, demonstrating its utility in tracking disease impact.

Additionally, explore the possibility of including bolt‐on additional items to address identified gaps in content coverage. These bolt‐on items could capture DMD‐specific concerns not adequately addressed by the standard EQ‐5D dimensions. The Y‐3L doesn't specifically address fatigue and energy levels, which are common symptoms in DMD. Although mobility is included, the tool may not fully capture the specific challenges related to muscle weakness and its impact on daily activities. Moreover, social participation, which can significantly affect quality of life, is not explicitly addressed. Cognitive and learning difficulties experienced by some paediatric patients are overlooked, as is the caregiver burden, which indirectly impacts the patient's well‐being. While anxiety and depression are considered, other emotional aspects, such as coping strategies and resilience, are not included. Addressing these gaps may enhance the tool's sensitivity, providing a more comprehensive assessment of HRQoL for patients with DMD. Furthermore, investigate correlations between EQ‐5D scores and DMD‐specific clinical measures to validate their ability to reflect clinically meaningful changes.

### 4.2 Limitations

Several limitations should be addressed. First, we admit that EQ‐5D‐5L was explored with greater thematic depth than the EQ‐5D‐Y‐3L, leading to an imbalance in evidence density that may weaken conclusions for the youth version. This likely results from children and adolescents with DMD providing fewer details than adults, possibly due to developmental differences, difficulties articulating HRQoL concepts, or reliance on caregiver proxies. In future research, we will use age‐appropriate qualitative methods, such as simplified interview guides or visual aids, to elicit more comprehensive responses from paediatric DMD patients, strengthening the evidence for the EQ‐5D‐Y‐3L. Second, we used web‐based interviews as the sole data collection method. Patients unfamiliar with this approach may have been excluded from recruitment, resulting in selection bias. Last, while our adult DMD patients ranged between 18 and 32 years of age—and we acknowledge that DMD significantly affects life expectancy—this age range means that the voices of older DMD patients could not be heard, limiting the generalisability of our findings.

## 5 Conclusion

This study demonstrates that both the EQ‐5D‐5L and Y‐3L exhibit acceptable content validity for assessing HRQoL in Chinese DMD patients, providing evidence for potential use in the future economic evaluations of health technologies, treatments and medicines for DMD. However, our findings highlight the need to refine the wording and structure of EQ‐5D instruments to better capture key domains relevant to DMD and potentially other neuromuscular diseases. Specifically, incorporating items that address mental health, peer communication and social support, which are areas where EQ‐5D instruments are known to have limitations, could enhance their relevance to patients' quality of life and well‐being. Future studies should evaluate the psychometric properties of EQ‐5D instruments alongside DMD‐specific PROMs across diverse cultural contexts and among patients with a wider range of demographic characteristics. This will enable a more comprehensive and accurate assessment of HRQoL in this population, ultimately supporting improved patient care, treatment evaluation and health policy decisions.

## Author Contributions

**Richard Huan Xu:** conceptualisation, methodology, software, validation, formal analysis, data curation, funding acquisition, writing – original draft, writing – review and editing. **Rui Jiang:** project administration, data curation, writing – review and editing. **Chenxi Yang:** project administration, writing – review and editing. **Dong Dong:** conceptualisation, supervision, formal analysis, writing – review and editing.

## Ethics Statement

The Institutional Review Board of Hong Kong Polytechnic University approved the research protocol (Ref. ID: HSEARS20230412007‐02).

## Consent

All the participants provided written informed consent.

## Conflicts of Interest

The authors declare no conflicts of interest.

## Supporting information


**Table a1:** Interview guideline for EQ‐5D‐5L. **Table a2:** Interview guideline for EQ‐5D‐Y‐3L. **Table a3:** Operational Definitions of EQ‐5D‐5L Dimensions for DMD. **Table a4:** Operational definition of EQ‐5D‐Y dimensions in DMD. Figure a1: Coding tree of assessing content validity of the EQ‐5D‐5L/Y.

## Data Availability

The data that support the findings of this study are available from the corresponding author upon reasonable request.
