# Supplementary material for: A Qualitative Examination of the Content Validity of the EQ‐5D‐5L and EQ‐5D‐Y‐3L in Adult and Paediatric Patients With Duchenne Muscular Dystrophy
Source: Health Expect. 2025 Sep 10;28(5):e70431. doi: 10.1111/hex.70431 (PMC12422114; doi:10.1111/hex.70431)
Supplement: Supplementary file 1 — Table a1: Interview guideline for EQ‐5D‐5L. Table a2: Interview guideline for EQ‐5D‐Y‐3L. Table a3: Operational Definitions of EQ‐5D‐5L Dimensions for DMD. Table a4: Operational definition of EQ‐5D‐Y dimensions in DMD. Figure a1: Coding tree of assessing content validity of the EQ‐5D‐5L/Y. [file HEX-28-e70431-s001.docx]

**Table a1 Interview guideline for EQ-5D-5L**

|  | Questions | Content |
| --- | --- | --- |
| Preparation | 1 | The interviewers introduce themselves and provide some background to this research |
|  | 2 | Declarations (e.g., informed consent, record the interview, etc.) |
|  | 3 | Collect informaiton about background (age, education background, living situation, and long-term conditions) |
|  | 4 | Talk briefly about your current QoL and factors that influence it |
|  | 5 | Complete the EQ-5D-5L |
| Interview | 6 | can you please have a look at these questions and tell me the initial impressions, including whether it was clear, easy to understand, and of acceptable length. |
|  | 7 | Let’s start by talking about the **physical aspect (including mobility and pain)** of having DMD. What effects does DMD have on you doing things with your body and your daily physical activities? |
|  | 8 | [Refer to the EQ-5D]. Do you think these questions ask about what you have just mentioned? |
|  | 9 | Let’s move on now to talk about how DMD **makes you feel (mental health part)**. How does having DMD make you feel? |
|  | 10 | [Refer to the EQ-5D]. Do you think this question ask about what you have just mentioned? |
|  | 11 | Let’s move on to talk about **doing usual acitivies** Does having DMD affect the things you can do for usual acitivies alone with your friends/others? |
|  | 12 | [Refer to the EQ-5D]. Do you think this question ask about what you have just mentioned? |
|  | 13 | Can we talk now about how you **feel about looking after yourself**? How does DMD affect these things? |
|  | 14 | [Refer to the EQ-5D]. Do you think this question ask about what you have just mentioned? |
|  | 15 | Are there any other ways that DMD affects your life? This could be anything that is important to you. |
|  | 16 | if you revise the scale, any health aspects that are important that can be added to the questionnaire? |

**Table a2 Interview guideline for EQ-5D-Y-3L**

|  | Questions | Content |
| --- | --- | --- |
| Preparation | 1 | The interviewers introduce themselves and provide some background to this research |
|  | 3 | Declarations (e.g., informed consent, record the interview, etc.) |
|  | 4 | Collect information about background (age, education background, living situation, and long-term conditions) |
|  | 5 | Talk briefly about your current QoL and factors that influence it |
|  | 6 | Complete the EQ-5D-Y |
| Interview | 7 | can you please have a look at these questions and tell me the initial impressions, including whether it was clear, easy to understand, and of acceptable length. |
|  | 8 | Let’s start by talking about the **physical aspect (including mobility and pain)** of having DMD. What effects does DMD have on you doing things with your body and your daily physical activities? |
|  |  | [Refer to the EQ-5D(Y)]. Do you think these questions ask about what you have just mentioned? |
|  | 9 | Let’s move on now to talk about how DMD **makes you feel (mental health part)**. How does having DMD make you feel? |
|  |  | [Refer to the EQ-5D(Y)]. Do you think this question ask about what you have just mentioned? |
|  | 10 | Let’s move on to talk about **doing fun things and your friends**. Does having DMD affect the things you can do for fun and with your friends and others? |
|  |  | [Refer to the EQ-5D(Y)]. Do you think this question ask about what you have just mentioned? |
|  | 11 | Can we talk now about how you **feel about yourself and doing things by yourself**? How does DMD affect these things? |
|  |  | [Refer to the EQ-5D(Y)]. Do you think this question ask about what you have just mentioned? |
|  | 12 | Are there any other ways that DMD affects your life? This could be anything that is important to you. |
|  | 13 | if you revise the scale, any health aspects that are important that can be added to the questionnaire? |

**Table a3 Operational Definitions of EQ-5D-5L Dimensions for DMD**

| **Dimension** | **Operational Definition for DMD** | **Rationale** |
| --- | --- | --- |
| Mobility | The ability to move oneself or navigate one’s environment, including walking, transferring (e.g., from bed to wheelchair), using a wheelchair, or performing compensatory movements with upper limbs or assistive devices. | Reflects progressive mobility loss in DMD and participant redefinitions emphasizing upper limb-assisted movements (e.g., joystick control for wheelchairs). |
| Self-Care | The ability to perform personal care tasks such as washing, dressing, feeding, or grooming, with or without assistance from adaptive devices, caregivers, or upper limb function. | Incorporates upper limb tasks (e.g., eating, grooming) critical for DMD patients, as highlighted by participants, especially in non-ambulatory stages. |
| Usual Activities | The ability to engage in daily activities relevant to the individual’s life, such as schoolwork, hobbies, social interactions, or work, with or without modifications, assistive devices, or support. | Accommodates diverse, age- and stage-specific activities meaningful to DMD patients, from education to adapted leisure activities. |
| Pain/Discomfort | The experience of physical pain or discomfort, including muscle or joint pain, contractures, or discomfort from prolonged sitting, immobility, or medical interventions (e.g., scoliosis surgery). | Captures DMD-specific sources of pain, such as muscle degeneration or postural issues, relevant to the EQ-5D-5L dimension. |
| Anxiety/Depression | The experience of emotional distress, including feelings of anxiety, sadness, or depression related to living with DMD, disease progression, social limitations, or caregiving needs. | Addresses psychological impacts of DMD, including concerns about disease progression and loss of independence. |

**Table a4 Operational definition of EQ-5D-Y dimensions in DMD**

| **Dimension** | **Operational Definition for DMD** | **Rationale** |
| --- | --- | --- |
| Mobility (Walking about) | Ability to move around, including walking, using a wheelchair, or upper limb movements. | Reflects DMD mobility challenges and participant redefinitions (e.g., upper limb use). |
| Looking after myself | Ability to wash, dress, eat, or brush teeth, with or without help or devices. | Includes upper limb tasks critical for DMD, age-appropriate for youth. |
| Doing usual activities | Ability to do everyday activities like school, play, or hobbies, with or without help. | Captures diverse, age-specific activities for young DMD patients. |
| Having pain or discomfort | Feeling pain or discomfort from muscles, joints, sitting, or treatments. | Addresses DMD-specific pain sources in simple language for children. |
| Feeling worried, sad, or unhappy | Feeling worried, sad, or unhappy about DMD, its progression, or social challenges. | Reflects emotional impacts of DMD, aligned with EQ-5D-Y phrasing. |


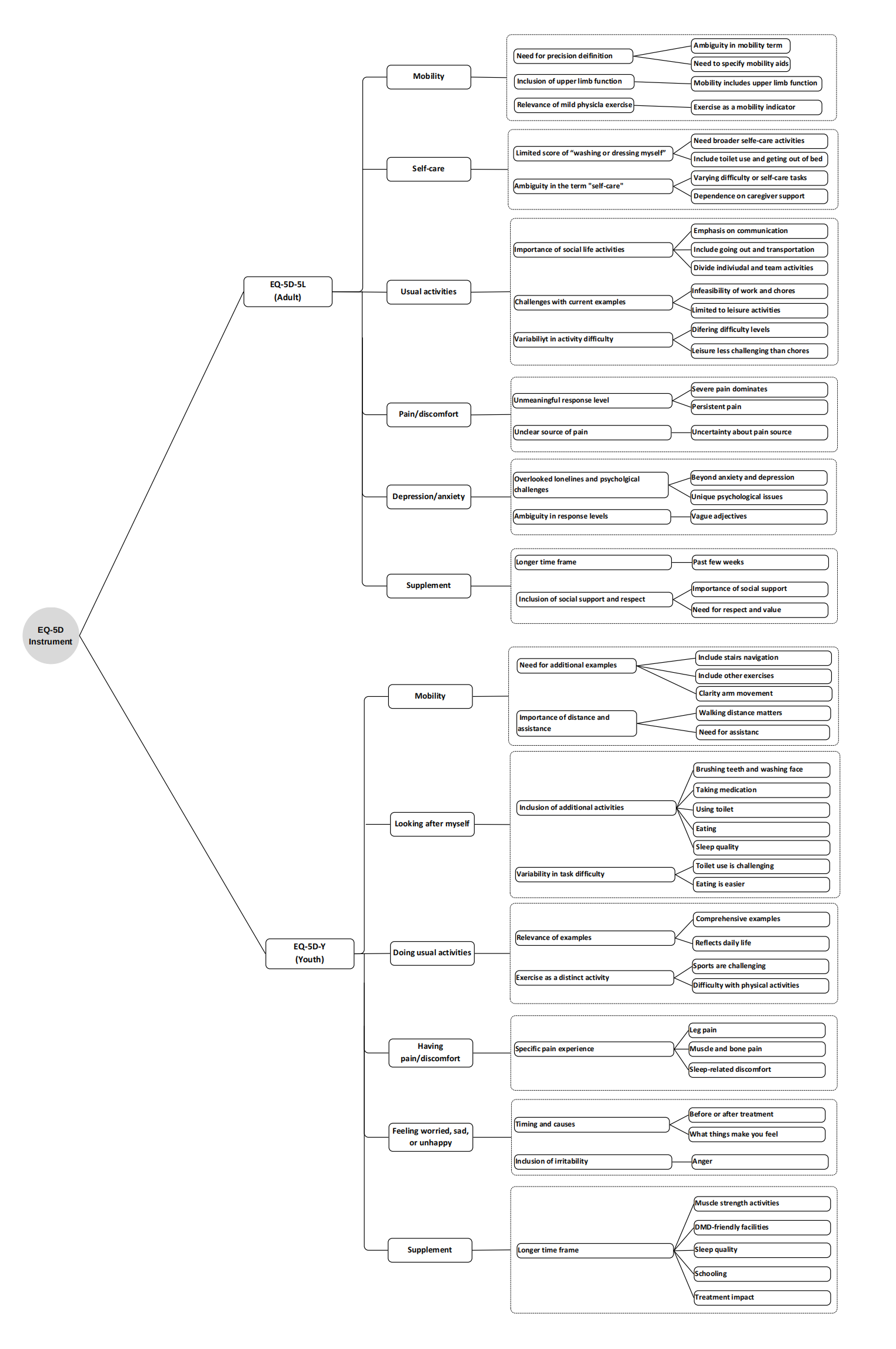


Figure a1 Coding tree of assessing content validity of the EQ-5D-5L/Y
